# Case Studies about Finite Element Modeling and Wireless Sensing of Three Pennsylvania Bridges

**DOI:** 10.3390/s24061714

**Published:** 2024-03-07

**Authors:** Alireza Enshaeian, Behzad Ghahremani, Piervincenzo Rizzo

**Affiliations:** 1 Department of Civil and Environmental Engineering, University of Pittsburgh, Pittsburgh, PA 15261, USA; ale69@pitt.edu; 2 Department of Mechanical Engineering and Materials Science, University of Pittsburgh, Pittsburgh, PA 15261, USA; beg66@pitt.edu

**Keywords:** bridge diagnostics, wireless sensors, physics-based structural health monitoring, strain analysis

## Abstract

Three Pennsylvanian bridges were studied using finite element and wireless sensor technology. A detailed finite element model of each bridge was created using a commercial software in order to calculate the strains generated by a load that simulates the presence of a standard truck. Pristine and damage scenarios were simulated, and the computed strains were compared to the experimental strains measured with proprietary wireless sensors during a truck test performed by companies not involved in the study presented in this article. The comparison demonstrated the accuracy of the model and the presence of a few non-critical anomalies in terms of load redistribution. In addition, the comparison proved the reliability of the wireless sensing system installed on the bridges, although some drift was observed. The structural monitoring program for the three bridges was also evaluated by processing more than two years of data streamed to a repository.

## 1. Introduction

In many countries, there is an increasing pressure on bridge owners to develop cost-effective strategies to prioritize maintenance, rehabilitation, and replacement and minimize inconveniences related to postings and traffic closures. In the United States, some Departments of Transportation use methodological frameworks to quantify the criticality of each asset and identify bridges whose intervention should be prioritized [1]. The occurrence of bridge failures or the need to diagnose critical assets in the aftermath of natural events, such as earthquakes, warrants the use of complementary approaches to supply timely information about any given bridge [2]. One element of concern is fatigue, especially in those bridges that typically have high live load to dead load ratios and high stress cycle frequencies [3]. One of the complementary approaches is offered by structural health monitoring (SHM) in which sensors of different kinds are bonded/bolted/embedded to the structure of interest and stream real-time measurements to a central repository where smart processing is performed with the goal of flagging critical issues as they unfold. As noted by Cawley [4], the replacement of periodic inspection of engineering structures with SHM systems has been discussed for many years. However, the industrial take-up of SHM technology has been slow due to organizational and business case issues as well as significant technical challenges [4]. Nonetheless, SHM, which sits at the crossroads of sensor technology, signal processing, and structural engineering, has seen rapid growth in various research [5,6,7,8]. One of the challenges in the application of SHM in bridges is the useful insights that can be obtained from the massive amount of “big data” [9]. 

Traditional bridge health monitoring in the United States is mostly based on the use of strain gages, accelerometers, and displacement sensors. A review by Rizzo and Enshaeian [10] showed that other devices include, but are not limited to, tiltmeters [11,12], weigh-in motion sensors [13], non-contact displacement measurements [14], corrosion sensors [15], and acoustic emission [16,17].

The health monitoring of bridges is often complemented by finite element (FE) modeling, which is sometimes accompanied by controlled truck load tests. For example, Li and Glisic [18] coupled 3D FE modeling with the SHM data to interpret the response of a steel–concrete composite scale model structure with minute damage. The model spanned 9.14 m and a width of 3.66 m, consisted of a 203 mm thick reinforced concrete slab connected to three W16 × 57 steel girders, and was monitored through a set of loading events. Svendsen [19] and Svendsen et al. [20] performed numerical and experimental studies of a full-scale steel bridge to establish an experimental benchmark to obtain response measurement data under different structural state conditions with imposed damage. He proposed a hybrid SHM framework for damage detection by integrating numerical models, experimental data, and machine learning. Park et al. [21] created an FE model for the Galena Creek Bridge, allegedly the largest concrete cathedral arch bridge in the world, located in Nevada. A detailed FE model was developed using CSiBridge software v22.1.0, and a parametric study was conducted to examine how a range of variables influenced the dynamic properties of the structure. Static dead load, modal, and time-history analyses were performed to provide insights regarding the influence of each parameter. Sharma et al. [22] compared the efficiency of two different types of sensors, i.e., accelerometers and strain gages, in identifying damage in a multi-span concrete box girder bridge. A high-fidelity finite element model of the bridge was created in CSiBridge. By incorporating a range of damage scenarios, they assessed the bridge’s response to the loading of a standard passing truck in both the time and frequency domains. Kaloop et al. [23] collected static and dynamic data from the Dorim-Goh Bridge and the Tun Bridge [24], both located in Korea, using ambient trucks and information in time and frequency domains. Another example is the work of Hedegaard et al. [25], who created a linear elastic finite element model of the concrete post-tensioned box girder I-35W St. Anthony Falls Bridge and validated it using truck-load tests. Other notable examples of bridges studied with FE in support of SHM systems are the Aizhai suspension bridge [26], the Nanjing Yangtze River Bridge [27], the Xiabaishi Bridge [28], the Tsing Ma Bridge [29], and a precast concrete bridge in the Dominican Republic [30]. It is noted here that a comprehensive review of existing bridge health monitoring strategies is outside of the scope of this paper. Interested readers are referred to Rizzo and Enshaeian [10] for a systematic review of the SHM programs of bridges in the United States. 

**Figure 1:**
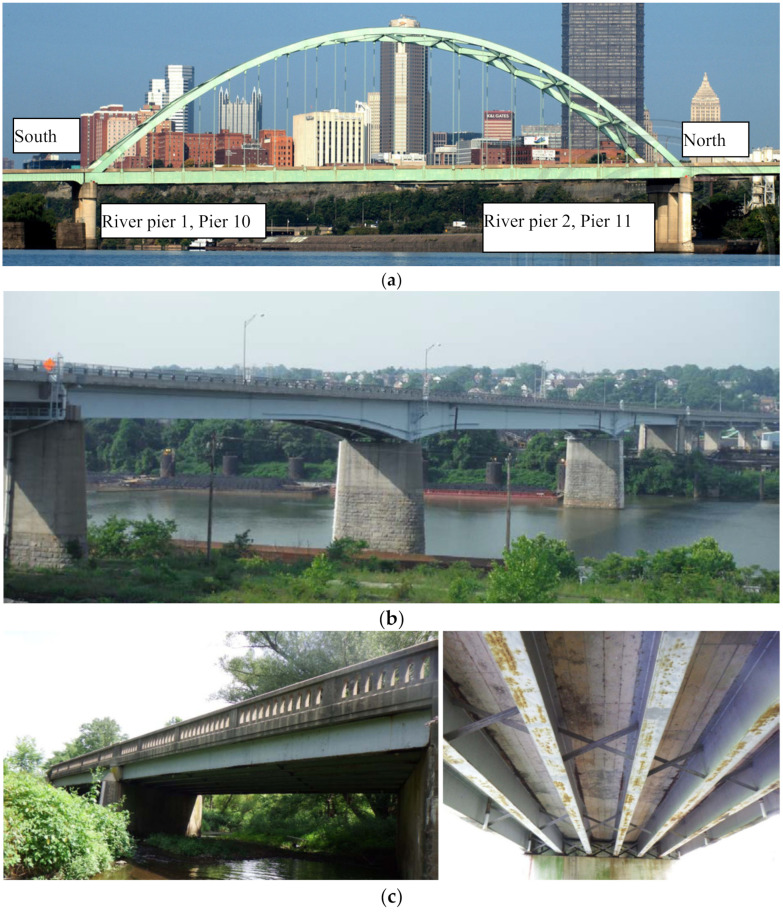
Photos of: (**a**) Birmingham Bridge (adapted from https://www.flickr.com/photos/jag9889/10148146816 (accessed on 14 January 2021)), (**b**) Glassport–Clairton Bridge, and (**c**) Chester Bridge. Upstream elevation looking downstream and deck underside and superstructure. Figure adapted from the 2015 bridge inspection report made available to the authors by the project sponsor.

In the study presented in this article, the three bridges shown in Figure 1 and located in southwestern Pennsylvania were modeled using ANSYS 2020 R2 software. For each bridge, a digital replica was created and a linear static analysis was applied to calculate the variation of the strains caused by a truck at specific locations on the structures. These numerical strains were compared against the experimental values recorded with an array of proprietary temperature-compensated wireless sensors during a truck test. The authors were informed that the bridges were closed to traffic while the test was taking place. Furthermore, the long-term monitoring data from the same sensors were processed to develop a SHM paradigm. The effect of transient events, such as heavy traffic, was extracted and separated from thermal effects and drift by computing the difference between the raw strains and the corresponding 15 min moving average. Hereinafter, such a difference is referred to as the true strain and shall not be confused with the actual strain as pre-stress, dead load, and the reference temperature at the time of sensor installation were not known to the authors. The hypothesis behind such a procedure is that the effect of any transient event, e.g., vehicle overload, vehicle crashes, or barge collisions, lasts much less than 15 min, and therefore its effect on the moving average of the strain is negligible. Meanwhile, the inertia of the steel is such that the deformation induced by the temperature takes longer than 15 min. The true strains associated with the individual sensors were then clustered to perform an outlier analysis (OA) based on the Mahalanobis squared distance (MSD) *D_ζ_*, defined as [31,32,33,34,35,36,37]:

(1) $$D_{\zeta }={\left(\left\{x_{\zeta }\right\}-\left\{\bar{x}\right\}\right)}^{T}\cdot {\left[K\right]}^{-1}\cdot \left(\left\{x_{\zeta }\right\}-\left\{\bar{x}\right\}\right)$$

Here, {*x_ζ_*} is the potential outlier vector, $\left\{\bar{x}\right\}$ is the mean vector of the baseline, [*K*] is the covariance matrix of the baseline, and *T* is the transpose symbol. 

The scientific contribution of this article is the systematic study of three different kinds of bridge by combining accurate finite element modeling, real long-term data, and a controlled truck loading test. As part of this study, this paper proposes a few simple strategies to identify structural anomalies of the structure as well as transient events. This paper expands the work of Ghahremani et al. [38], where the finite element analysis of a prestressed concrete box beam bridge was discussed. It is disclosed here that the installation of the sensing system and the truck load test were performed a few years ago by two companies not involved in this study. In addition, the raw data were downloaded from a password-protected repository. Access to this repository was granted by the Pennsylvania Department of Transportation, which sponsored this project. 

## 2. The Structures

The first bridge is the iconic Birmingham Bridge (Figure 1a), located in the city of Pittsburgh and completed in 1977. It comprises twelve spans, one of which is a bowstring arch crossing over the Monongahela River. The total length of the structure is 507 m (1662 ft), while the arch, which is the subject of this study, is 189 m (620 ft) long. Suspender cables support the deck of the steel-tied-arch, designated as Span 11 and standing on Piers 10 and 11 (River Piers 1 and 2). The second structure (Figure 1b) is the Clairton–Glassport Bridge that carries the traffic across the Monongahela River between Glassport and Clairton Counties south of Pittsburgh. The bridge was completed in 1987 and rehabilitated in 2011 and consists of steel girders and prestressed concrete I-beams. Overall, the structure has 17 spans, for a total length of 668 m (2190 ft). Spans 11 through 13 across the river are the subject of this study. The last structure (Figure 1c) is the two-spans BMS ID 15-0082-0592-0385 bridge, which carries the traffic of Manor Road over East Branch Brandywine Creek in Chester County. This rolled steel I-Beams bridge was built in 1937, and its total length is 105 ft (32 m). This bridge is hereinafter referred to as the Chester Bridge. 

## 3. Instrumentation and Truck Test Setup

**Figure 2:**
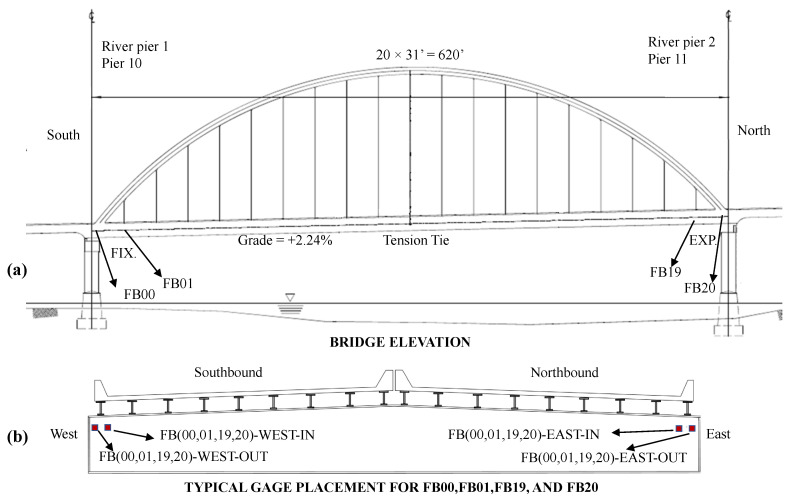
Instrumentation plan of the Birmingham Bridge. (**a**) Elevation view from East. (**b**) Cross-section view from south side. The figures were adapted from instrumentation plans provided to the authors by the project sponsor.

The Birmingham Bridge was instrumented with 18 displacement sensors, 17 tiltmeters, and 16 strain gage rosettes. The displacement sensors were positioned at the bearing locations to record relative longitudinal movement between the superstructure and the substructure. The rosettes were installed on two sides of the copes (i.e., the notch in the floor beams at their connection to the tension tie) of the floor beams FB00, FB01, FB19, and FB20, as shown in Figure 2. The rosettes were installed on the lower corners on each side. The rosette closer to the tension tie is labeled with the suffix “OUT” while the rosette at the opposite side of the cope is labelled with the suffix “IN”. The rosettes enabled the measurement along the vertical (XX), transverse (YY), and 45° (XY) directions. A truck test was performed using a 69,960 lb truck. The steer axle was 19,040 lb and the drive “tandem” axles were 50,920 lb. The test lasted two hours and consisted of 18 crossings, with 9 northbound and 9 southbound. For each bound, the truck travelled on the bike, right, center, and left lanes. One crossing included a stop on the right lane close to the floor beams FB00, FB01, FB19, and FB20. The test lasted about 2½ h.

**Figure 3:**
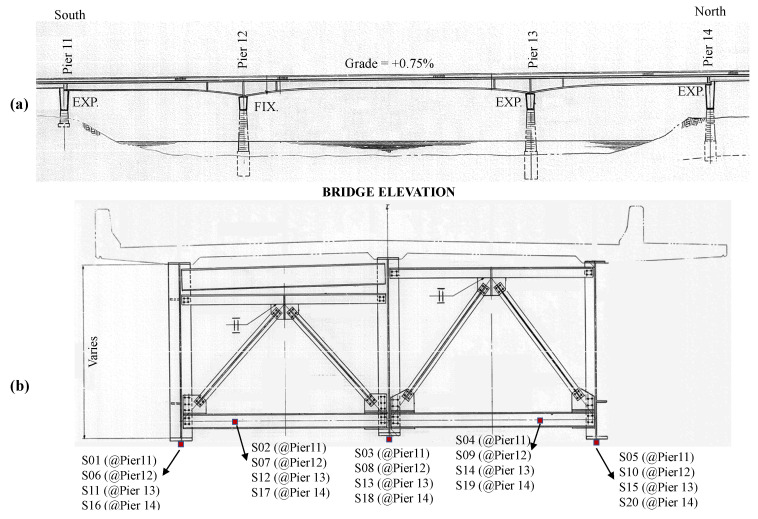
Instrumentation plan of the Clairton Bridge according to the information provided by the project sponsor. (**a**) Elevation view. (**b**) Visualization of the cross-section.

**Figure 4:**
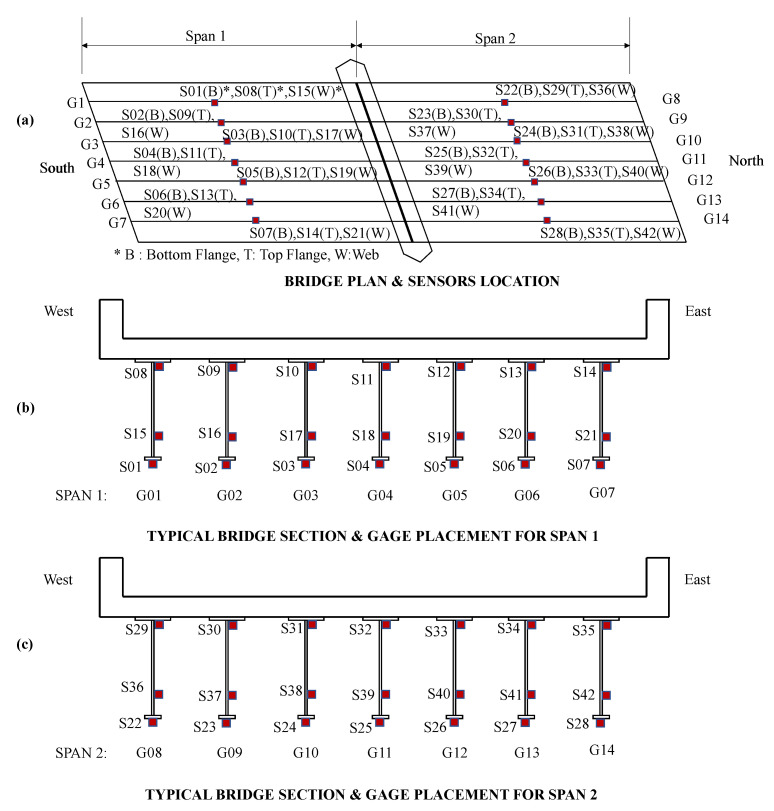
Instrumentation plan of the Chester Bridge according to the information provided by the project sponsor. (**a**) Bridge plan and location of the sensors at both spans of the bridge. (**b**) Bridge section of span 1 and placement of the wireless strain gages. (**c**) Bridge section of span 2 and placement of the wireless strain gages.

The Clairton Bridge was instrumented with 20 strain gages, 24 displacement sensors, 4 two-dimensional tiltmeters, 4 accelerometers, and 4 cameras at locations shown in Figure 3. Three cameras aimed to capture barge collisions impact at Piers 2, 3, and 4, respectively. The displacement gages were placed at the bearings to record either the longitudinal or the transverse movement between the superstructure and substructure. There was no live load truck test performed. Finally, forty-two strain gages were bonded to the Chester Bridge according to the layout of Figure 4. A truck test was performed using a 58,460 lb truck. The steer axle was 18,400 lb and the drive “tandem” axles were 40,060 lb. The protocol consisted of crossing the bridge at 8 km/h and 48 km/h northbound and southbound, crossing at 8 km/h along the middle of the bridge, and then stopping the truck at the center of Span 1 and Span 2 along the north and the bound lane. The test lasted two hours.

## 4. Modeling: Setup and Simulated Damage

**Figure 5:**
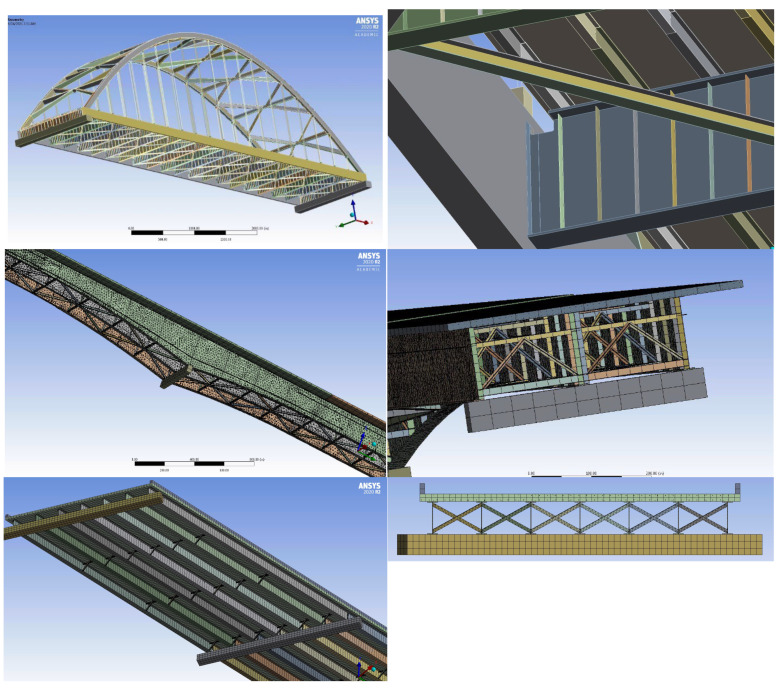
Snapshots of the detailed finite element models of the three bridges considered in this study. (**Top row**): Birmingham Bridge. (**Center row**): Clairton–Glassport Bridge. (**Bottom row**): Chester Bridge.

The finite element replica of the Birmingham Bridge (Figure 5) consisted of 8124 structural components, 452,000 finite elements, and about 1,310,000 nodes. Most components were considered solid bodies, whereas line bodies were used for the cables and for the rebar. The material properties are summarized in Table 1 and are representative of the properties selected for the other two bridges as well. For the Clairton Bridge, class AAA cement concrete was used for the slabs, whereas ASTM A36 [39], A572 [40], and A588 [41] were used for the steel elements. The model included 5242 structural components and about 285,800 finite elements and 835,300 nodes. The replica of the Chester Bridge consisted of 441 structural components, more than 90,900 finite elements, and over 400,700 nodes. It is noted here that for every bridge, the largest number of structural components was represented by the steel reinforcement of the concrete deck. 

The original (pristine) conditions were simulated by considering the shop drawings provided to the authors by PennDOT. Then, the six damage scenarios summarized in Table 2a–c were implemented individually. To mimic any loss of composite behavior on the Chester Bridge, the contact type of the girders was changed from “Bounded” to “No Separation”. In ANSYS 2020 R2 software, “Bounded” contact between two objects means that the objects are perfectly bounded to each other, and they cannot slide or be separated. “No separation” contact type between two components means that the objects can slide along the contact surface, but they cannot be separated.

**Figure 6:**
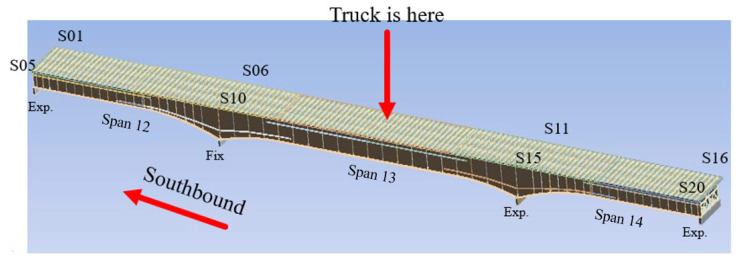
Clairton Bridge analysis. Schematics of Load Case 2: the load of a truck in the southbound direction is applied to the middle of Span 13.

For the Birmingham and the Chester Bridge, a static analysis was conducted by applying a six-point load on the deck to reproduce the action of the truck’s steering wheels and tandem axles. Owing to the lack of truck test, the static analysis of the Clairton Bridge consisted of applying the weight of a 58,460 truck (steer axle and drive tandem axle equal to 18,400 lb and 40,060 lb, respectively) at the center of the northbound direction of Span 12 and at the center of the southbound direction of Span 13 (Figure 6). 

## 5. Truck Test Results and Comparison to the Numerical Analyses

### 5.1. Birmingham Bridge

**Figure 7:**
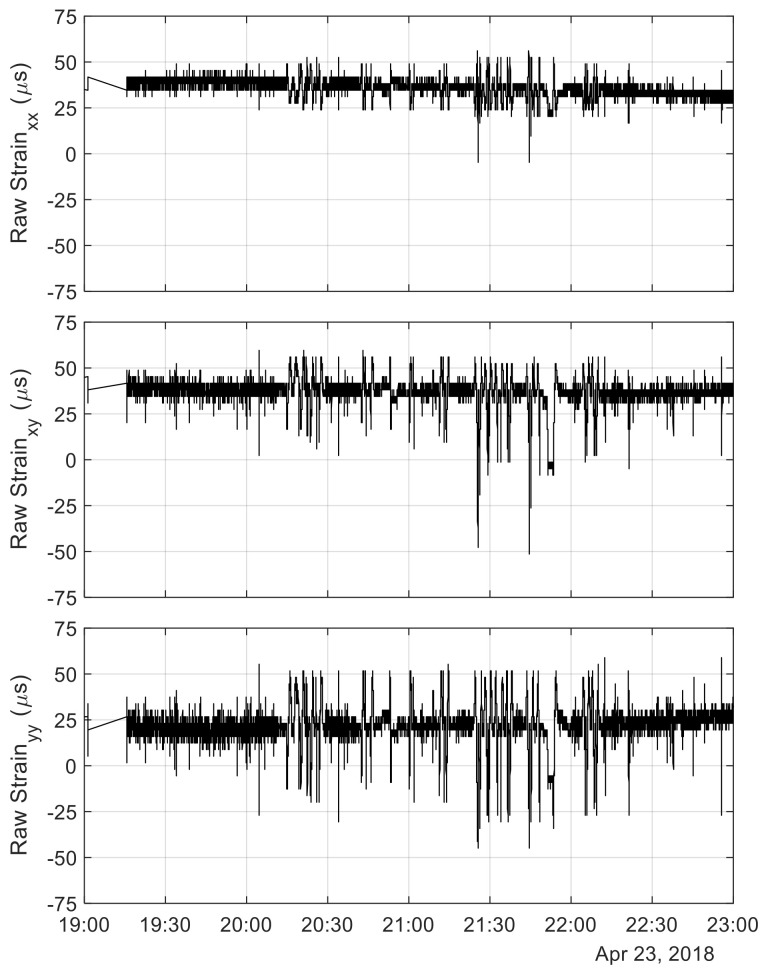
Representative result of the truck load test for the Birmingham Bridge. Strain readings from rosette FB01-WEST-IN along (**top** to **bottom**) the vertical, 45 degrees, and horizontal directions, respectively. The horizontal axis represents the date and the hours during which the test was performed.

For illustrative purposes, Figure 7 shows the raw strains recorded by the rosette FB01-West-IN. The label indicates that the sensor was on the west side of floor beam 1, farther from the tension tie. The number of twin-spikes between the hours 20:00 and 22:15 is consistent with the 18 truck crossings. The baseline strain, i.e., the measurement without the truck, remained nearly constant, as the temperature of the steel did not change much during the experiment. 

**Figure 8:**
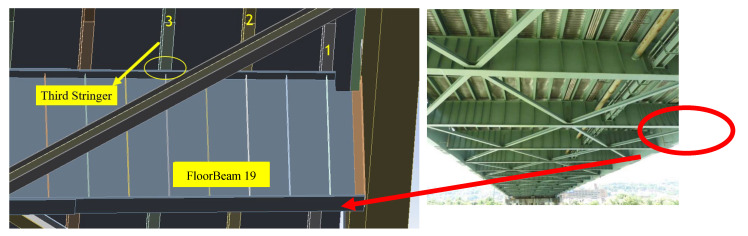
Location of the simulated damage of Scenario 3 on the Birmingham Bridge.

The results of the truck test were compared to the numerical calculations obtained with the ANSYS model. Table 3 lists the results associated with two specific truck crossings and the computed strains relative to the original bridge design and to the bridge under Damage Scenario #3. This damage consisted of locking the contact of stringer No. 3 with floor beam 19, as seen in Figure 8. 

**Figure 9:**
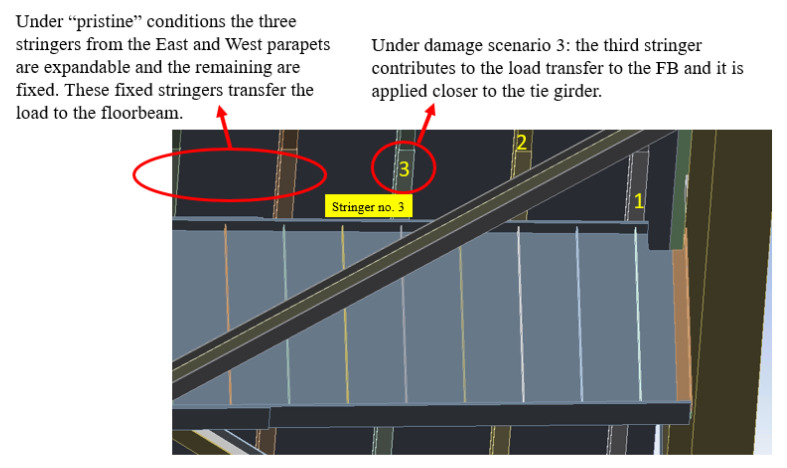
Birmingham Bridge. Load transfer mechanism between stringer 3 and FB19 under pristine conditions and Damage Scenario 3. Note that the numbers on the figure label the stringer number.

Table 3a lists the maximum vertical (XX) and horizontal (YY) strains recorded by the four rosettes located on the east side of the bridge while the truck crossed the northbound bike lane (twice). Note that if E = 29,000 ksi, the value of 100 με corresponds to 2.9 ksi of stress. The sensors did not peak simultaneously. For example, the maximum YY strain at FB19-EAST-IN (equal to −79.4 με) did not occur when sensor FB19-EAST-OUT experienced its maximum value of −12.7 με. When this was the case, an asterisk on Table 3 indicates the strains recorded at the same instant. This choice enables the comparison of the numerical calculations computed with a truck at a specific bridge location and the consequent deformation across the neighboring sensors. Table 3b lists the results associated with the rosettes located on the west side of the bridge when the truck crossed the southbound right lane (twice). The experimental values listed in the table were extracted from the tables reported by the company responsible for the truck test and made available to the authors of this article by the project sponsor. The asterisks label those values extrapolated from the strain waveforms presented in the mentioned report. Table 3 shows that the experimental strains recorded by the sensors that are equidistant from the centerline of the bridge did not deform evenly. For example, the FB01-East-IN rosette responded differently during two identical crossings (−73.40 με vs. −57.5 με). Similar considerations can be made for FB19-West-Out along the YY direction (22.00 με vs. 28.00 με). This mismatch may be attributed to experimental uncertainties, including the fact that the sensors are bonded to areas of stress concentration. Despite the complexity of the structure, the numerical results under pristine conditions overall agreed well with the experimental values. The rightmost column of each graph quantifies the percentage difference between the original design and the numerical damaged scenario. For the inner gages, the variation in the predicted strains may be significant. The origin of such variation is illustrated in Figure 9, which explains the difference between the load transferring from the stingers to the floor beams under pristine conditions and under Damage Scenario 3. The third stringer contributed to the load transfer to the floor beams; therefore, some of the truck load was shifted toward the tie girder. Thus, the damage caused more compression at the locations of inner sensors. Similarly, according to the FE model’s results presented in Table 3, the inner sensors in YY direction showed more compression.

When the modulus of elasticity of steel was reduced by 5% (Damage Scenario 1), the strains uniformly increased by about 5%. Similar outcomes were observed when the modulus of elasticity of the concrete deck was reduced by 10%, as a 1% increase was found, showing that the change in concrete stiffness did not much change the deformation at the sensors’ location. One of the simulations included the removal of four cables: one inner cable from the east side above FB01, one inner cable from the west side above FB01, one inner cable from the east side above FB19, and one inner cable from the west side above FB19. The cable removal reduced the bending moment at the connection of the floor beam to the tie girder similarly to a change in the boundary condition from quasi-“fixed” support to quasi-“pin” support. The removal of the cable increased the load carried by the adjacent cables by 15–20%. However, this kind of damage may not be detected by the installed sensors. So, the location of the sensors is pivotal to detect this kind of damage. The same conclusions were drawn after analyzing the results relative to Damage Scenarios 5 and 6.

### 5.2. Clairton–Glassport Bridge

**Figure 10:**
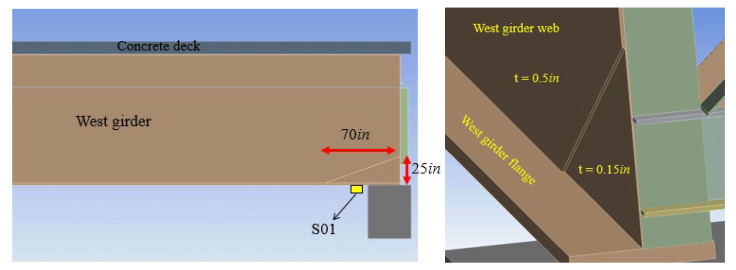
Clairton Bridge modeling. Damage Scenario 5: side view and 3D view from the end of the west girder.

For illustrative purpose, Figure 10 shows the location of Damage 5. The web thickness of the west girder was reduced from 0.5 inches to 0.15 inches due to triangle-shaped corrosion at the south end of the bridge (Span 12). Table 4 presents the results. As the damage was just above S01, its larger effect was detected by that sensor, whereas it is negligible elsewhere. The S01 strain was 31% lower, which seems to be counterintuitive as a thinner web should induce larger localized stress and strain. As a matter of fact, the effect of the same damage at other locations of the west girder increased the strain at the middle of the west girder. As such, the 31% strain reduction reported in Table 4 may be the effect of local instances that are not well-captured by the model given that the values are quite small. 

**Figure 11:**
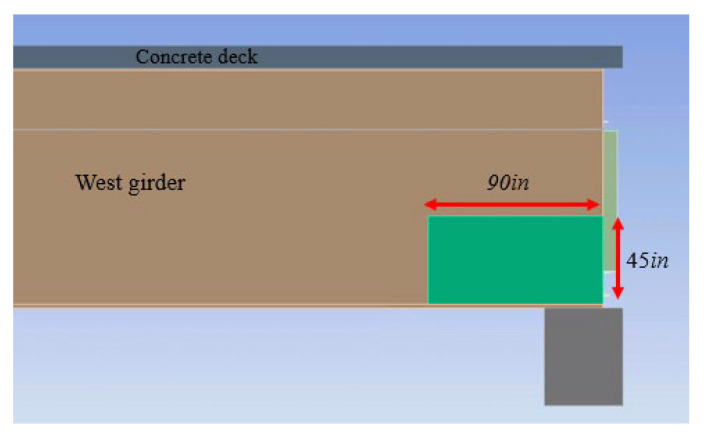
Clairton Bridge modeling. The added rectangular plate to the location of the fifth damage scenario.

The analyses associated with the other damages showed the following. The reduction of the modulus of elasticity of the steel (case scenario 1) increased the overall strains by about 5%, consistent with the linearity of the model and the fact that most members of the bridge are made of steel. When the modulus of the concrete deck was reduced by 15% (case scenario 2), the strains were 1.5% higher than the original design. This modest increase was driven by the dominance of the steel elements. The removal of the diaphragms and diagonal bracings from the middle of Span 12 (west side) and Span 13 (east side) did not affect the overall strains at the sensors’ locations. This is compatible with the fact that the simulated damage was too far away from the sensors. This was proven by evaluating the strains at the middle of Span 13. Under Load Case 2, strain difference is in the order of 4–6%, proving that sensors’ locations are a key factor in the success of any health monitoring program. When the modulus of elasticity of the middle girder was reduced by 10% (Damage 4), the girders experienced about 2–3% higher strain. Because the damage changed the stiffness of the middle girder, load distribution between the girder changed. The last simulation on the Clairton Bridge consisted of adding a 0.5-inch rectangular plate to the location of the previous damage scenario in order to mimic a repair (Figure 11). The subsequent analysis demonstrated that the repair reinstated the pristine condition of the girder.

### 5.3. Chester Bridge

**Figure 12:**
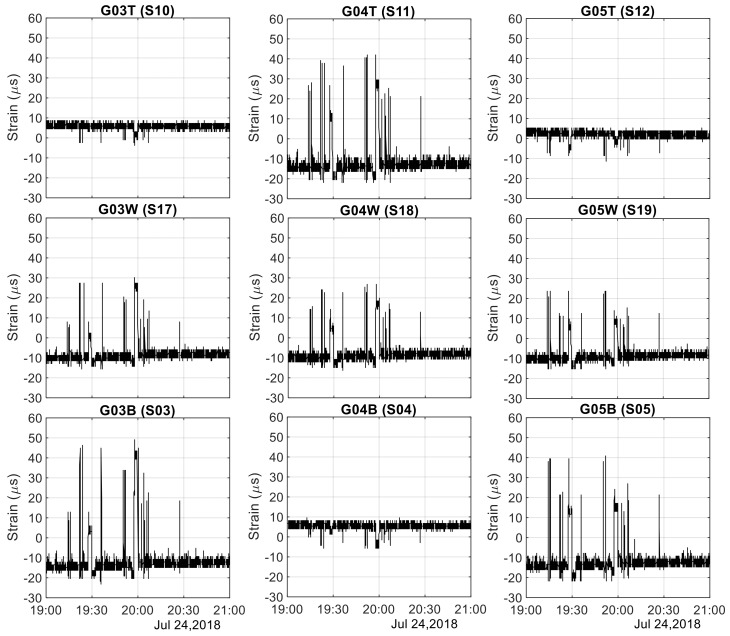
Chester Bridge truck testing. Raw strains recorded by the sensors bonded to Girder 3 (**left column**), Girder 4 (**middle column**), and Girder 5 (**right column**). From top to bottom, the sensors were those bonded to the top flange, the web, and the bottom flange. The horizontal axis represents the date and the hours during which the test was performed.

The neutral axis of this steel–concrete composite bridge is localized close to the top flange. Figure 12 shows the raw strains recorded by the gages bonded to Girders 3, 4, and 5 during the truck test. While the response at the web was quite similar, the measurements at the top and bottom flanges across the three girders were uneven. After reviewing the technical report submitted by the company performing the truck test, it was determined that the repository mislabels the time-series. As such, the time-series labeled as G04B is to be intended as G04T and vice versa. With that clarification, the subplots in Figure 12 demonstrate that Girders 3 through 5 worked as a composite bridge. Under the effect of the truck load, the top flange underwent compression and the bottom flanges experienced larger tensile stress than the web.

**Figure 13:**
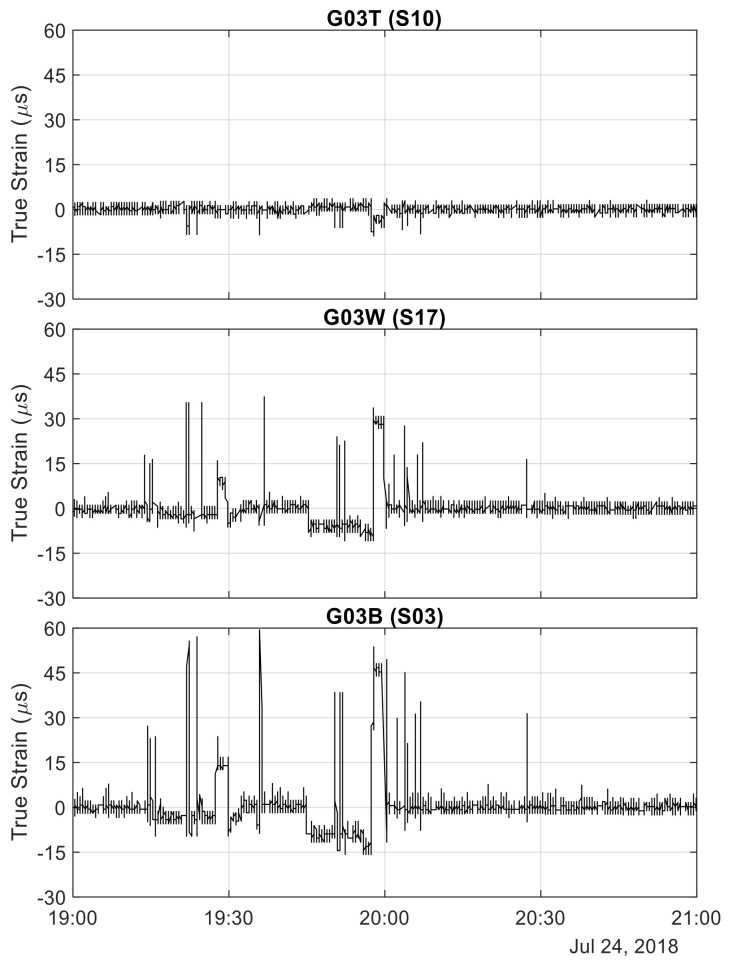
Representative result of the truck load test on the Chester Bridge. True strains relative to three strain gages. The horizontal axis represents the date and the hours during which the test was performed.

The graphs of the true strains (Figure 13) prove that the thermal-related bias is removed while keeping the information relative to the strain increase caused by the truck. As a matter of fact, the values of the peaks are not uniform, consistent with the fact that the truck crossed the bridge at different lateral distances, i.e., above different girders.

**Figure 14:**
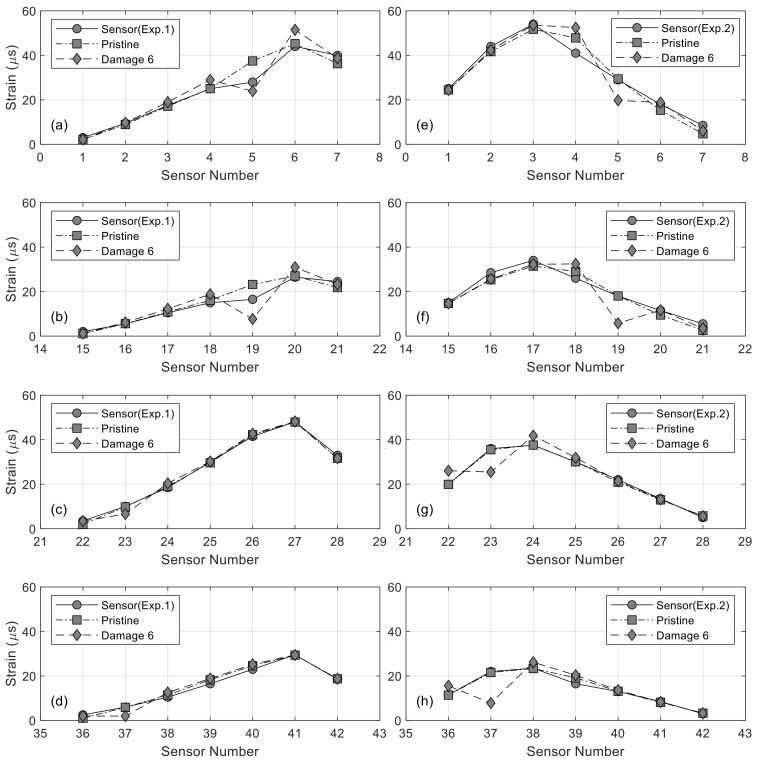
Chester Bridge. The predicted strains at certain locations of the bridge for the sixth damage scenario. (**a**–**d**) Refer to Experiment 1, and (**e**–**h**) refer to Experiment 2. In Exp. 1 and 2, the truck travelled over the bridge northbound and southbound, respectively. For (**a**,**b**,**e**,**f**), the truck is at the middle of the first span, and for (**c**,**d**,**g**,**h**), it is at the middle of the second span. The field data were extracted from the graphs published by the company executing the test in the report provided to the authors by the project sponsor.

The empirical values of the Chester Bridge were compared to the computed strains. Specifically, Figure 14a–d present the case of the truck standing above the center of Span 1 and Span 2 of the northbound lane, whereas Figure 14e–h refer to the truck standing at the southbound lane. The experimental and numerical strains under pristine conditions and Damage Scenario 6 are presented. The measured strains agreed extremely well with the FE results. The few exceptions of S4, S5, and S19 are attributed to uneven transfer across adjacent members as proven by looking at the difference between pristine conditions and damaged scenarios.

Regarding the other scenarios, it was found that the corrosion of the bottom flanges (Damage 1), which represented about 15% thickness reduction, increased the strain by 6–7%, as the neutral axis shifts. Under Damage Scenario 2, where the thickness of the web decreased by 10% from 0.564 in to 0.504 in, the strains increased about 3–4%. Owing to the linearity of the model, a 5% decrease in the Young’s modulus of the steel (Damage #3) increased the strain by about 5%. The localized damage at Girders 3 and 12 (Damage #4) induced some local change in the predicted strain, as can be seen in Table 5. In this damage scenario, the modulus of elasticity reduction was applied for only two beams. The results show that all of the beams experienced an increase in strains. Because the beams are connected to each other through the deck, the adjacent beams bore some of the effect of G3 and G12 deterioration. The absence of seven diaphragms (steel bracing) that mimic the localized rupture of some welded connection between the girders and diaphragms yielded to very modest differences in strains, with a few exceptions visible in Table 6. As the diaphragms connect two adjacent beams at three single points, they did not contribute to load distribution significantly, and the deck was mainly responsible for load distribution. Therefore, the simulated damage did not alter the strain values at the web or bottom flange of the girders. 

## 6. Long-Term Monitoring

### 6.1. Raw Strain Data

**Figure 15:**
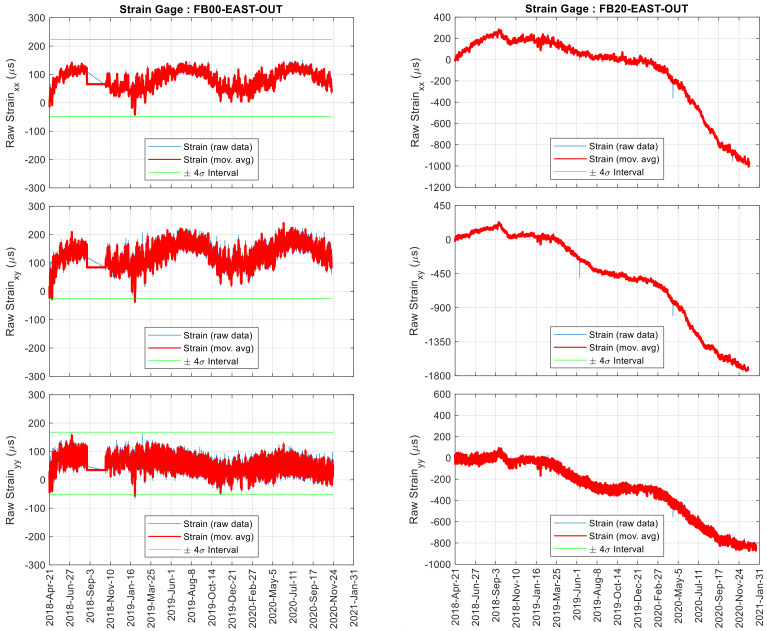
Raw and moving averaged strain recorded through 31 January 2021, Birmingham Bridge. The horizontal axis represents the monitoring timeframe considered in this study.

**Figure 16:**
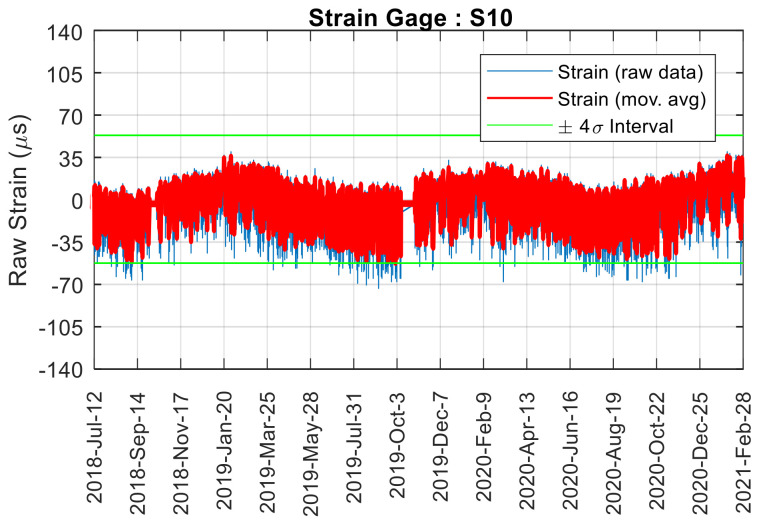
Raw and moving averaged strain recorded through 28 February 2021, Clairton Bridge. The horizontal axis represents the monitoring timeframe considered in this study.

Representative historical raw strains and the corresponding 15 min average for the Birmingham Bridge are given in Figure 15. While the rosette bonded to the floor beam labelled as 00 had a seasonal cyclic trend, the gage bonded on its symmetric counterpart (FB20) drifted. It is noteworthy that a few other sensors mounted on the Birmingham Bridge experienced drift. Figure 16 presents a representative time waveform of the Clairton Bridge strain. With respect to the Birmingham Bridge, the temperature-related variations are lower, likely because of the element being monitored. The repository lacked data around October 2019 for reasons unknown to the authors.

The Chester Bridge was monitored for 2 ½ years. For the sake of space, no time histories are presented here. It was observed that most of the gages displayed a clear cyclic trend associated with the seasons. The sensors bonded to Girders #1 and #8 exhibited larger daily variations than any other girders because they were exposed to the west. Five sensors stopped working earlier than the others. 

### 6.2. Raw Strain–Temperature Analysis 

**Figure 17:**
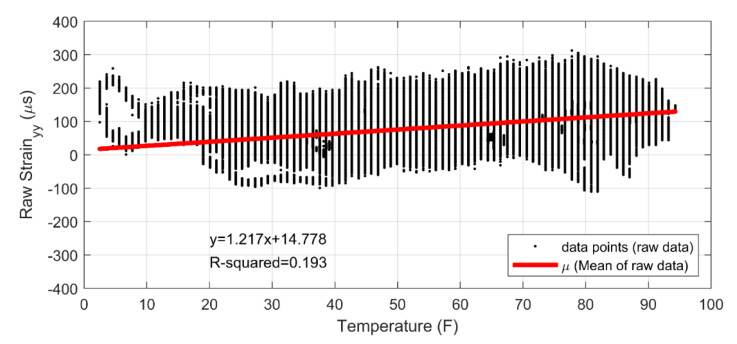
Birmingham Bridge. Raw strain vs. raw temperature recorded by gage FB00-WEST-IN along the yy direction. The period of observation lasted until 31 January 2021.

**Figure 18:**
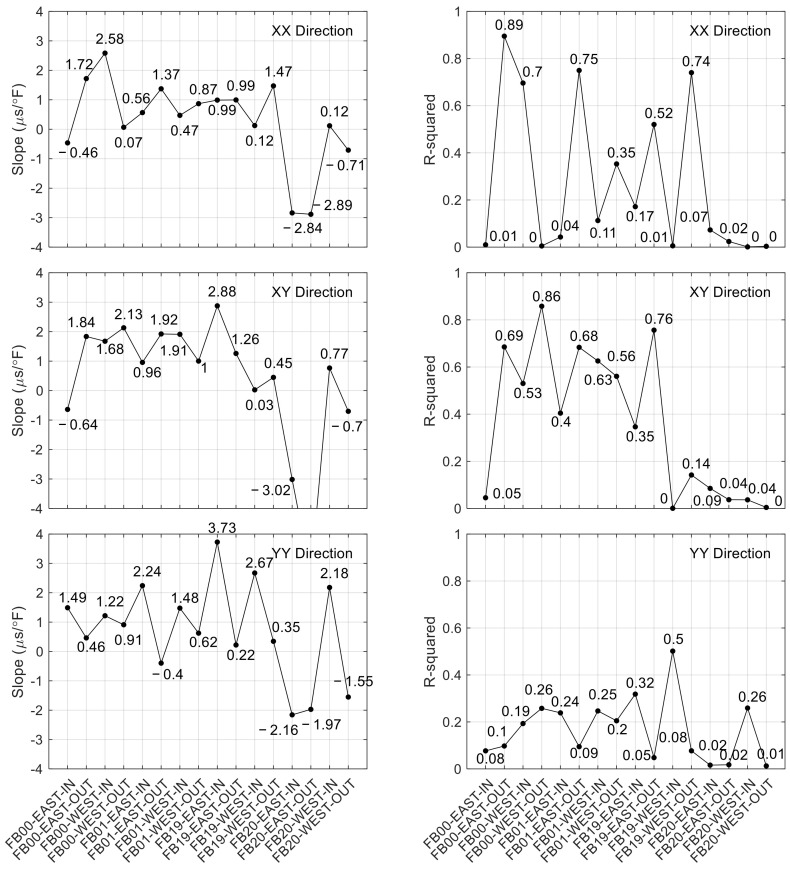
Birmingham Bridge. Slope of linear regression of the strain vs. temperature graphs (the values are expressed in µε/°F) and residual R^2^ of the linear interpolation strain vs. temperature. The horizontal axis indicates the names of the sensors considered in the graph.

The deformation of the structures caused by the ambient temperatures was investigated as a possible marker for structural anomalies. To describe the procedure, Figure 17 presents the raw strains and the associated temperature from the Birmingham Bridge. The equation of the linear regression and the corresponding R^2^ values are overlapped. The strain gradient, namely the slope of the regression, and the R^2^ from all of the rosettes bonded to the Birmingham Bridge are displayed in Figure 18. Most of the gradients are positive, which means that the structural member expanded with the increase in the temperature. The two evident exceptions were the two rosettes bonded on the east side of FB20. As those sensors drifted, their reliability is questionable. Looking at the residuals, those associated with the direction perpendicular to traffic (YY) were lower than the vertical and diagonal directions. Most of the gages with the lowest R^2^ (<0.1) also exhibited the slope closest to zero, which implies that the corresponding structural member was not prone to significant thermal effects. The behavior of the gages bonded to symmetric members was not necessarily similar. For example, the slopes associated with FB00-EAST-IN and FB00-WEST-IN were equal to −0.46 με/°F and 2.58 με/°F (along XX), respectively, and −0.64 με/°F and 1.68 με/°F (along XY), respectively.

**Figure 19:**
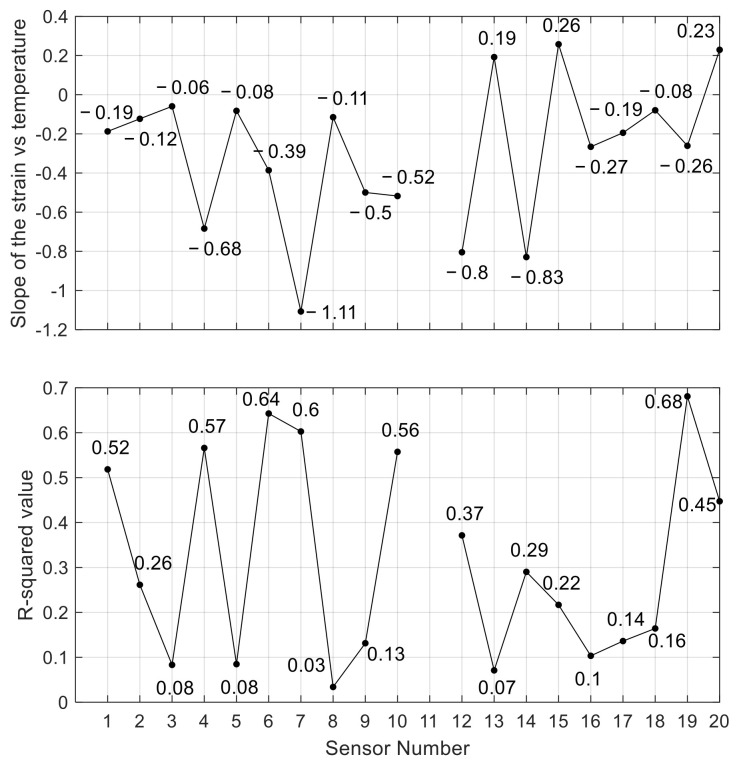
Clairton Bridge. Slope of linear regression of the strain vs. temperature graphs (the values are expressed in µε/°F) and residual R^2^ of the linear strain vs. temperature.

**Figure 20:**
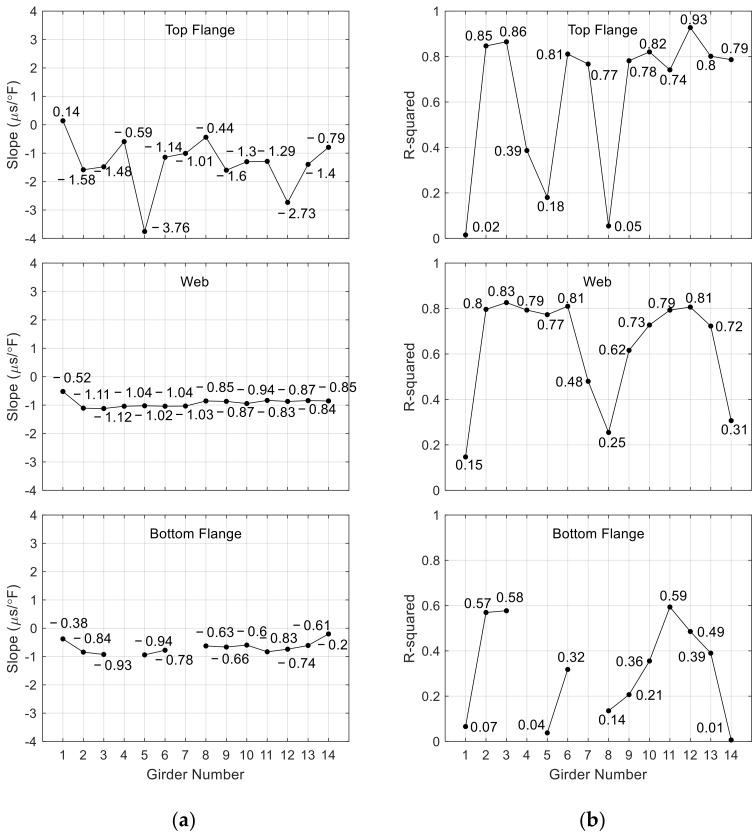
(**a**) Slope of linear interpolation of the strain vs. temperature graphs. The values are expressed in με/°F. (**b**) Residual R^2^ of the linear interpolation strain vs. temperature.

A similar analysis was carried out for the other two bridges, and the results are presented in Figure 19 (Clairton Bridge) and Figure 20 (Chester Bridge), respectively. Gages S04, S07, S12, and S14 bonded to the Clairton Bridge have the largest negative gradients. These sensors are bonded to the web of the lower beam of diaphragms (see Figure 3b), which are therefore highly responsive to temperature changes. Additionally, it is evident that even within a single diaphragm, the sensitivity to temperature is not consistent. For instance, although sensors S07 and S09 are both installed on the web at Pier 12, the slope relative to S07 is more than twice that of S09. Moreover, their R^2^ values differ significantly. This inconsistency between these two sensors can be partially attributed to the asymmetric geometry of the diaphragm (Figure 3b). In contrast, S04 and S19, located at the southern and northern piers (Piers 11 and 14), respectively, appear to provide consistent strain data. The R^2^ values are nearly the same (around 0.6). In addition, the strain–temperature slopes for these two gages indicate that they are not highly responsive to temperature changes.

The results of the Chester Bridge are clustered according to the girders’ numbers. Girders 1 through 7 belong to Span 1, and Girders 8 through 14 belong to Span 2. Some data are not included because the corresponding sensors stopped working too early. The gradients calculated from the sensors bonded to the top flange are not uniform, with an obvious discordance noted at Girder 5. Notably, the two girders facing west had the lowest residual. The thermal response at the webs was uniform and clearly linear. The only exceptions were the external girders (1, 7, 8, and 14). Being located close to the neutral axis, the web sensors were less prone to bending stress. Finally, regarding the bottom flange, the slopes associated with the inner girders, i.e., 2 through 6 (Span 1) and 8 through 13 (Span 2) were overall uniform and between −0.93 and −0.60 με/°F. The negative sign suggests that the increase in the temperature compressed the bottom flange, i.e., negative bending. Despite being located on the same west side of the bridge, the deformation of Girder 1 was nearly half that of Girder 8. As noted in Figure 14, the residual seen for the bottom flange was quite low when compared to the others. 

### 6.3. Live Load Analysis

**Figure 21:**
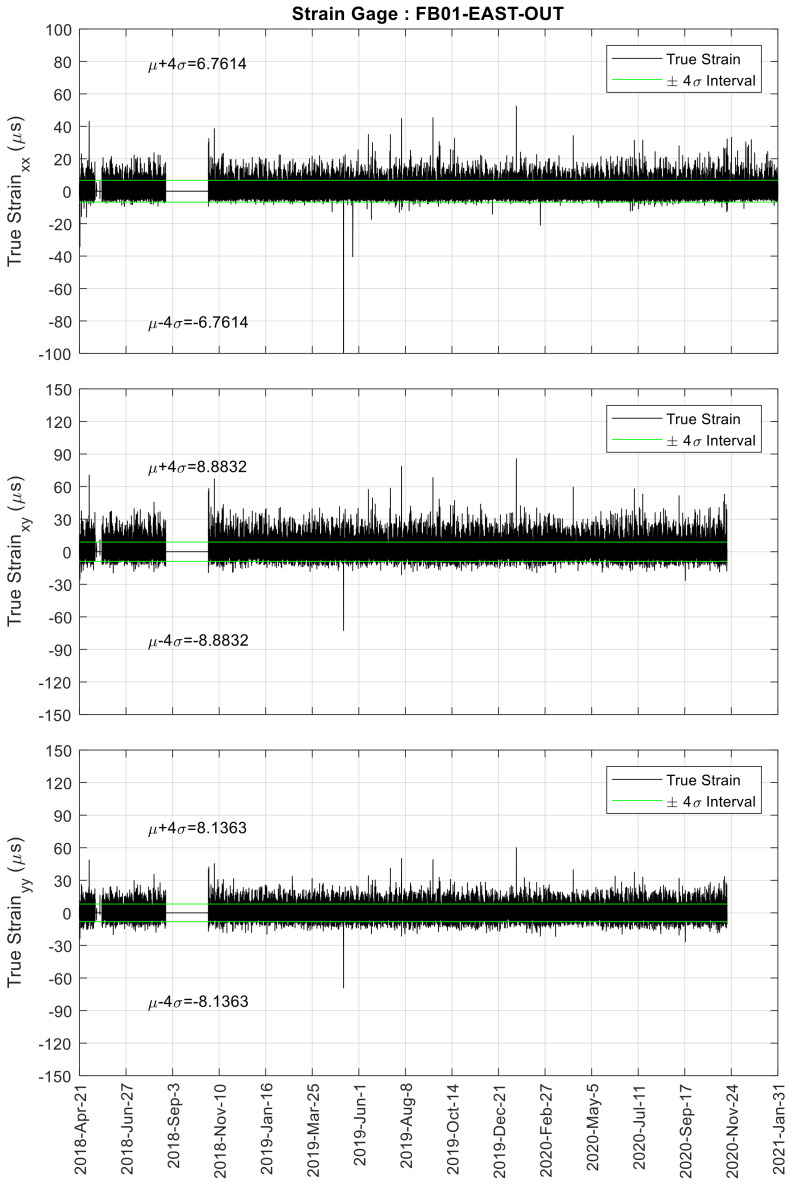
Brimingham Bridge. Time–histories of the calculated true strains from the rosette FB01-EAST-OUT. The horizontal axis represents the monitoring timeframe considered in the graph.

**Figure 22:**
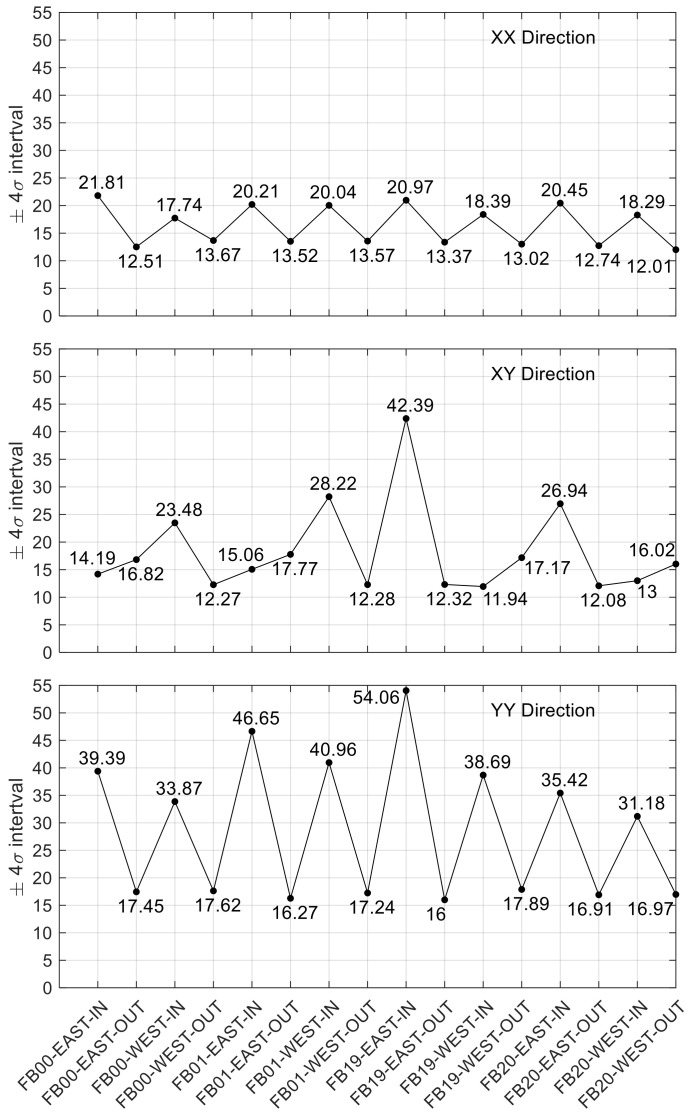
Birmingham Bridge, with ±4σ intervals associated with the cleansed true strains. The horizontal axis indicates the name of the sensors considered in the graph.

A representation of the true strain computed from FB01 of the Birmingham Bridge is provided in Figure 21. Each time-series was completed with the superposition of two horizontal lines that represent the strain range ±4σ. A few abnormal peaks were seen in spring 2019, and their origin could not be determined, although it may be the effect of the simultaneous crossing of heavy trucks. Besides the identification of spikes attributable to transient events, graphs like those shown in Figure 22 can be used to ascertain the responsiveness of the bridge to traffic loadings. A quantitative measure could be the estimation of the ±4σ interval. This analysis is presented in Figure 22. When comparing the graphs from the other strain gage, the north side of the bridge seems to experience more traffic load than the south side. Overall, the rosettes bonded on the outer side of the cope were less subjected to the effect of traffic. Interestingly, the sensing system on the south side of the bridge reported intervals that were 10–20% higher than the south side. One possible reason is related to the road network. There are two ramps close to the north side (Pier 11) whereas vehicles travel at full speed when close to the south pier (Pier 10).

The values of the ±4σ intervals computed from the rosettes are presented in Figure 22. The comparison of the ±4σ across symmetric elements or across adjacent members may be interpreted as a marker of the structural response to traffic load, the responsiveness of each sensor, and the deformation of the monitored structural member. Figure 22 shows that the ±4σ intervals relative to the YY direction are twice as high as those of the corresponding XX direction. The sensor bonded on the north and east sides of the bridge on floor beam #19 shows the highest interval. It is worth noting that any ±4σ interval below 8 με is compatible with readings that fluctuate between + and − 1 με, which is the resolution of the gages according to the manufacturer.

**Figure 23:**
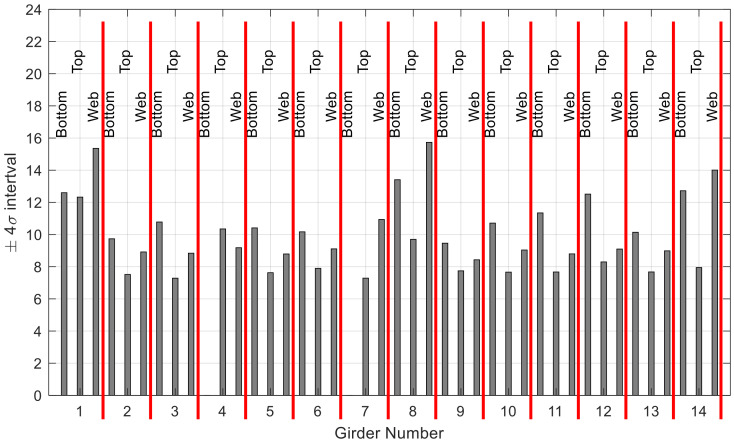
Value of the ±4σ interval in με calculated for each strain gage. Chester Bridge.

The same analysis was carried out for the other two structures. For the Clairton Bridge, the analysis did not reveal anything significant. The ±4σ interval for most gages was below 10 με, i.e., within the resolution of the sensors. The results relative to the Chester Bridge (Figure 23) show that the largest ±4σ intervals are associated with the sensors bonded to the web of Girders 1, 8, and 14, which are located on the outer side of the bridge (east side or west side). Figure 23 also shows that most of the sensors bonded to the top flange had values below 8 με. None of the forty values reported stood out from the others, suggesting that all of the elements being monitored responded normally. 

**Figure 24:**
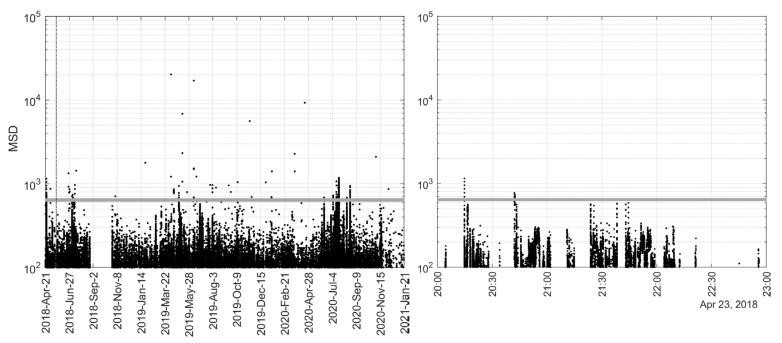
Outlier analysis of the Birmingham Bridge: XX-direction strains. Mahalanobis squared distance calculated during the whole monitoring period and during the truck test. The horizontal axis of the left plot represents the monitoring timeframe considered in the graph. The horizontal axis of the right plot represents the date and the hours of the truck test.

**Figure 25:**
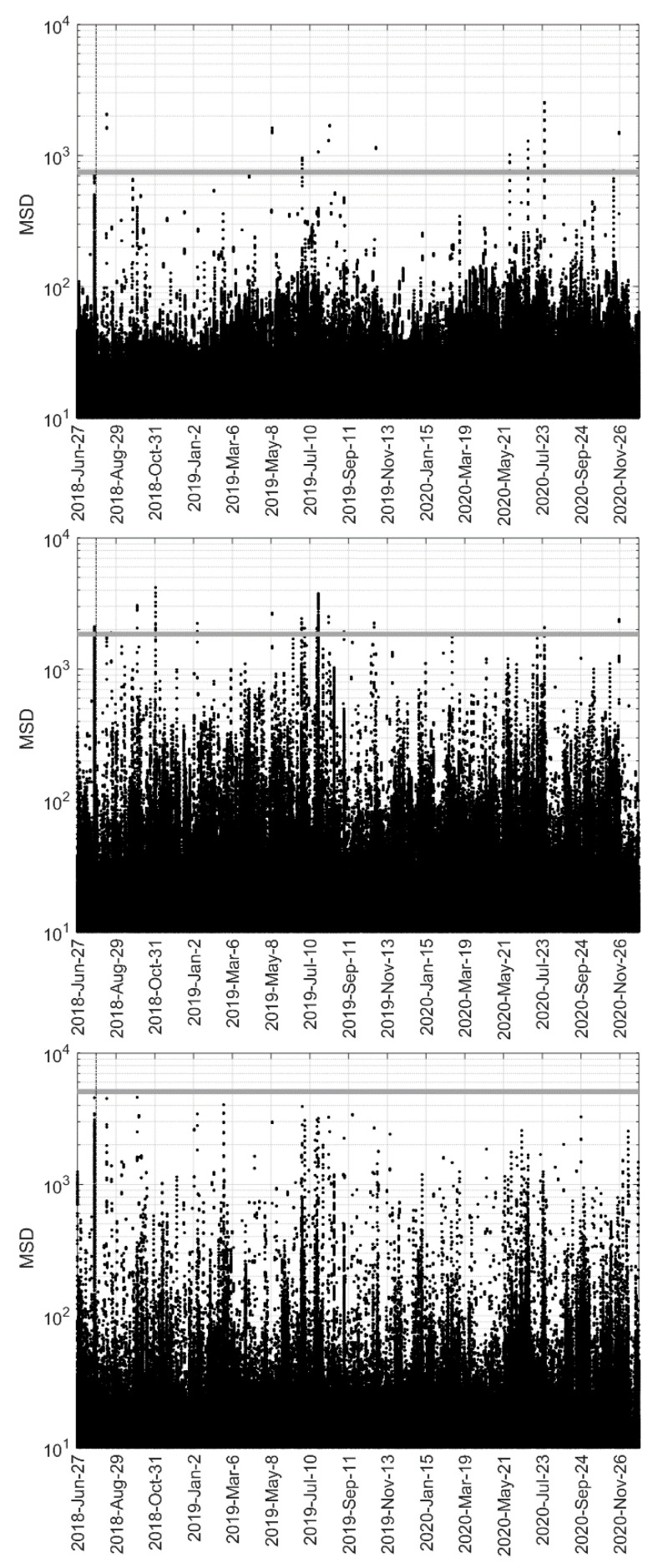
Mahalanobis squared distance applied to the whole monitoring period of the Chester Bridge. (**Top row**): top flange. (**Middle row**): web. (**Bottom row**): bottom flange. The threshold was computed by considering the data from the first month of operation without the truck test. The horizontal axis of the left plot represents the monitoring timeframe considered in the graph.

The results of the OA are presented in Figure 24 and Figure 25. The first month of monitoring constituted the baseline to calculate the threshold. Figure 24 refers to the Birmingham Bridge and shows the MSD calculated for the truck test data and for the whole monitoring period. To ease comparison, the vertical axes are the same. The case of the XX-direction strain data is presented. The crossing of the truck did induce a few outliers, the first of which was likely related to the arrival of the crew at the bridge. In the investigation of the datapoints with the highest MSD values, it was found that the first four highest ones corresponded to the data recorded by FB19-EAST-OUT, FB20-EAST-OUT, and FB01-EAST-OUT. It is believed, but it is not proved with any experimental evidence, that the larger, sparse MSD values are related to the simultaneous crossing of more than one truck on this six-lane bridge. 

The MSD relative to the truck load test performed on the Chester Bridge did show only one outlier from the web data slightly above the threshold. The analysis applied to the whole dataset is presented in Figure 25. As the sensors bonded to the top flange were close to the neutral axis, the MSD threshold is about one order of magnitude smaller than the threshold associated with the sensors at the bottom flange. Overall, the strain gages bonded to the top flange and the web identify some outliers, including those discussed in the previous section. There were no outliers from strain gage data from the bottom flange, although some are very close to the threshold and of similar values to the truck test.

## 7. Conclusions

This article presented three case studies relative to the health monitoring of three bridges with different structural characteristics. The monitoring strategy included the numerical model of the three superstructures using an accurate finite element model and the analysis of the data streamed from arrays of wireless strain gages bonded to those three bridges by a company not involved in the study presented in this study. The strain measurements relative to a truck load test and the measurements collected during more than two years of structural monitoring were downloaded from a password-protected repository and processed. The interpretation of the experimental data was supported with a high-fidelity finite element model implemented using commercial software. The results showed the excellent agreement between the numerical and the field data. Such agreement validated the accuracy of the model. The comparison also revealed the presence of a few non-critical anomalies in one of the structures being monitored. 

For the Birmingham Bridge, some analyses conducted in this study but not presented here for the sake of space showed some mismatches for a few sensors, such as FB01-West-In and FB01-West-Out sensors, during some truck crossings. In a few cases, these mismatches between the experimental data and the ANSYS predictions under pristine conditions could be justified by the simulated damage scenarios. For example, Damage Scenario 3 (locked stringer) can justify the differences in FB01-West-IN and FB19-West-IN sensors along the horizontal direction. The installation of sensors at locations prone to high strain/stress concentration may also cause possible differences between experimental and numerical data. The analysis of the Clairton Bridge did not reveal anything relevant in terms of critical elements. The results of the static analysis of the Chester Bridge showed that the simulated damage scenarios rarely caused more than 4 µε of strain increase for the sensors. This evidence indicates that the sensitivity of the installed sensors is not sufficient to capture some structural deteriorations. Overall, the numerical strains associated with the undamaged bridge are more aligned with the measured data in comparison to the simulated damage scenarios. Any mismatch did not seem to be correlated with any of the damage scenarios simulated in this task. Finally, while most of the sensors operated well throughout the monitoring period considered in this study, some gages showed signs of drift and a few others stopped working earlier than expected, and the repository had two gages mislabeled.

## Figures and Tables

**Table 1:** Properties of the concrete and steel components used in the finite element models.

**CONCRETE**	Deck or slab	4000 psi
**STEEL**	Cables (only for Birmingham Bridge)	Grade 250 steel
	Other steel components (beams, girders, etc.)	Grade 60 steel

**Table 2:** (**a**) Damage scenarios simulated for the Birmingham Bridge. (**b**) Damage scenarios simulated for the Clairton Bridge. (**c**) Damage scenarios simulated for the Chester Bridge.

Damage Scenario	Description
(**a**)	
1	The modulus of elasticity of steel was reduced by 5%.
2	The modulus of elasticity of concrete was reduced by 10%.
3	The contact of the stringers (the third counting from the curbs) with FB01 and FB19 changed from expandable to fixed (locked).
4	Simultaneous absence of one of the inner cables above FB01 and FB19
5	Two diaphragms that are approximately under the front axle of the truck were removed.
6	Bolt loosening at the connection of FB01 to the west tie girder (the tie girder that is at the right-hand side of the southbound direction).
(**b**)	
1	The modulus of elasticity of steel was reduced by 5%.
2	The modulus of elasticity of the concrete deck was reduced by 15%.
3	Removed diaphragms and diagonal bracing members from the middle of Span 12 (west side) and Span 13 (east side).
4	The modulus of elasticity of the middle girder was reduced by 10%.
5	Beam end severe corrosion is modeled.
6	Repair Scenario 1: a rectangular plate was added to the location of the previous damage scenario to repair the modeled corrosion.
(**c**)	
1	A total of 0.1 inches of corrosion at the bottom flange of each girder.
2	A total of 0.06 in corrosion at the web of each girder.
3	The modulus of elasticity of the girders’ steel was reduced by 5%.
4	Damage Scenario 4: the modulus of elasticity of the steel for Girder 3 (G3) and Girder 12 (G12) was reduced by 10%.
5	Seven diaphragms (steel bracing) were removed.
6	The contact of Girder 5 (G5) and Girder 9 (G9) with the deck was changed from “Bounded” to “No separation” (loss of composite behavior).

**Table 3:** (**a**) Birmingham Bridge. The predicted strains at certain locations of the bridge for the third damage scenario. The measured data refer to the northbound crossing of the truck along the bike lane (two crossings were completed). (**b**) Birmingham Bridge. The predicted strains at certain locations of the bridge for the third damage scenario. The measured data refer to the southbound crossing of the truck along the right lane (two crossings were completed). Note that the values followed by an asterisk were extrapolated from the strain waveforms presented in load test report made available by the sponsor of the project.

(**a**)										
	**Strain in XX Direction (με)**					**Strain in YY Direction (με)**				
	**Measured**		**Predicted**			**Measured**		**Predicted**		
**Sensor No.**	**Truck Crossings**		**Pristine**	**Damage 3**	**Diff. (%)**	**Test 1**	**Test 5**	**Pristine**	**Damage 3**	**Diff. (%)**
FB01-East-IN	−73.40	−57.50	−62.72	−61.10	−2.6%	−81.40	−71.10	−76.61	−88.29	15.2%
FB01-East-Out	16.20	19.30	14.08	14.24	1.1%	14.70	18.40	19.22	20.43	6.3%
FB19-East-IN	−50.40	−49.50	−60.91	−61.92	1.7%	−79.40	−77.50	−72.67	−76.09	4.7%
FB19-East-Out	15.60	18.30	15.19	15.04	−1.0%	12.30 *	11.90 *	20.53	19.95	−2.8%
(**b**)										
	**Strain in XX Direction (με)**					**Strain in YY Direction (με)**				
	**Measured**		**Predicted**			**Measured**		**Predicted**		
**Sensor No.**	**Truck Crossings**		**Pristine**	**Damage 3**	**Diff. (%)**	**Test 11**	**Test 15**	**Pristine**	**Damage 3**	**Diff. (%)**
FB01-West-IN	−16.10	−16.20 *	−20.34	−20.01	−1.6%	−32.00 *	−29.10 *	−17.15	−27.03	58%
FB01-West-Out	19.00	19.00	14.34	14.03	−2.2%	19.70	20.10	23.49	22.56	−4.0%
FB19-West-IN	−15.40 *	−14.90 *	−19.83	−18.42	−7.1%	−34.50 *	−31.10 *	−21.80	−35.06	61%
FB19-West-Out	17.90	18.40	15.74	15.47	−1.7%	22.00	28.00	24.56	24.14	−1.7%

**Table 4:** Clairton Bridge. The predicted strains at certain locations of the bridge under Damage Scenario 5 and Load Case 2.

Sensor No.	Predicted (με)		Diff. (%)
	Pristine	Damage 5	
S01	4.88	3.36	−31.1%
S03	4.70	4.72	0.4%
S05	4.86	4.88	0.4%
S06	−16.42	−16.42	0.0%
S08	−12.65	−12.65	0.0%
S10	−7.60	−7.59	−0.1%
S11	−13.60	−13.61	0.0%
S13	−11.33	−11.33	0.0%
S15	−8.34	−8.34	0.0%
S16	2.79	2.79	0.0%
S18	2.36	2.36	0.0%
S20	2.71	2.70	−0.4%

**Table 5:** Chester Bridge. Predicted strains at certain locations under Damage Scenario 4 (Test 5).

Truck is at the Middle of the First Span				
Sensor No.	Measured Strain (με)	Predicted Strain (με)		Diff. (%)
		Pristine	Damage 4	
1	3.00	2.09	2.17	3.8%
2	9.50	8.91	9.11	2.2%
3	17.50	17.12	17.51	2.3%
4	25.00	25.04	25.58	2.2%
5	28.00	37.47	37.56	0.2%
6	44.00	45.23	45.25	0.0%
7	40.00	36.28	36.31	0.1%
15	2.00	1.08	1.13	4.6%
16	5.50	5.59	5.73	2.5%
17	10.50	10.87	11.09	2.0%
18	15.00	16.08	16.37	1.8%
19	16.50	23.20	23.24	0.2%
20	26.50	27.04	27.05	0.0%
21	24.50	21.81	21.80	0.0%

**Truck is at the Middle of the Second Span**				
**Sensor No.**	**Measured Strain (με)**	**Predicted Strain (με)**		**Diff. (%)**
		**Pristine**	**Damage 4**	
22	3.50	2.02	2.01	−0.5%
23	10.00	9.78	9.89	1.1%
24	18.50	18.98	19.30	1.7%
25	30.00	29.62	30.24	2.1%
26	41.50	42.26	43.61	3.2%
27	48.00	47.93	48.91	2.0%
28	33.00	31.65	32.04	1.2%
36	2.50	1.03	1.03	0.0%
37	6.00	5.88	5.93	0.9%
38	10.50	11.44	11.62	1.6%
39	16.50	18.25	18.66	2.2%
40	23.00	24.58	25.46	3.6%
41	29.50	29.36	29.98	2.1%
42	18.50	18.74	18.94	1.1%

**Table 6:** Chester Bridge. Predicted strains at certain locations under Damage Scenario 5 (Test 5).

Truck is at the Middle of the First Span				
Sensor No.	Measured Strain (με)	Predicted Strain (με)		Diff. (%)
		Pristine	Damage 5	
1	3.00	2.09	1.97	−5.7%
2	9.50	8.91	8.83	−0.9%
3	17.50	17.12	17.13	0.1%
4	25.00	25.04	25.39	1.4%
5	28.00	37.47	37.94	1.3%
6	44.00	45.23	45.44	0.5%
7	40.00	36.28	35.96	−0.9%
15	2.00	1.08	1.02	−5.6%
16	5.50	5.59	5.55	−0.7%
17	10.50	10.87	10.92	0.5%
18	15.00	16.08	16.33	1.6%
19	16.50	23.20	23.50	1.3%
20	26.50	27.04	27.27	0.9%
21	24.50	21.81	21.52	−1.3%

**Truck is at the Middle of the Second Span**				
**Sensor No.**	**Measured Strain (με)**	**Predicted Strain (με)**		**Diff. (%)**
		**Pristine**	**Damage 5**	
22	3.50	2.02	1.93	−4.5%
23	10.00	9.78	9.69	−0.9%
24	18.50	18.98	19.03	0.3%
25	30.00	29.62	29.74	0.4%
26	41.50	42.26	42.67	1.0%
27	48.00	47.93	48.04	0.2%
28	33.00	31.65	31.54	−0.3%
36	2.50	1.03	0.97	−5.8%
37	6.00	5.88	5.81	−1.2%
38	10.50	11.44	11.48	0.4%
39	16.50	18.25	18.33	0.4%
40	23.00	24.58	24.91	1.3%
41	29.50	29.36	29.50	0.5%
42	18.50	18.74	18.66	−0.4%

## Data Availability

Data are contained within the article.
